# Intraspecific Variation and Environmental Determinants of Leaf Functional Traits in *Polyspora chrysandra* Across Yunnan, China

**DOI:** 10.3390/plants14192953

**Published:** 2025-09-23

**Authors:** Jianxin Yang, Changle Ma, Longfei Zhou, Qing Gui, Maiyu Gong, Hengyi Yang, Jia Liu, Yong Chai, Yongyu Sun, Xingbo Wu

**Affiliations:** 1 College of Landscape Architecture and Horticulture Sciences, Southwest Forestry University, Kunming 650224, China; 2 Southwest Research Center for Engineering Technology of Landscape Architecture (State Forestry and Grassland Administration), Kunming 650224, China; 3 Yunnan Academy of Forestry and Grassland, Kunming 650201, China; 4 Yuanmou Desert Ecosystem Research Station, Kunming 650233, China; 5 State Key Laboratory of Efficient Production of Forest Resources, Beijing 100091, China; 6 Tengchong Forestry and Grassland Technology Extension Station, Tengchong 679100, China

**Keywords:** leaf functional traits, *Polyspora chrysandra*, environmental factors, PLS-SEM, intraspecific variation

## Abstract

Plant functional traits (PFTs) serve as key predictors of plant survival and adaptation to environmental gradients. Studies on intraspecific variation in PFTs are crucial for evaluating species’ adaptation to projected climate change and developing long-term conservation strategies. This study systematically investigated PFT responses in *Polyspora chrysandra* (Theaceae, Yunnan, China) through an integrated multivariate analysis of 20 leaf functional traits (LFTs) and 33 environmental factors categorized into geographical conditions (GCs), climate factors (CFs), soil properties (SPs), and ultraviolet radiation factors (UVRFs). To disentangle complex environmental–trait relationships, we employed redundancy analysis (RDA), hierarchical partitioning (HP), and partial least squares structural equation modeling (PLS-SEM) to assess direct, indirect, and latent relationships. Results showed that the intraspecific coefficient of variation (CV) ranged from 7.071% to 25.650%. Leaf tissue density (LTD), specific leaf area (SLA), leaf fresh weight (LFW), leaf dry weight (LDW), and leaf area (LA) exhibited moderate intraspecific trait variation (ITV), while all other traits demonstrated low ITV. Reference Bulk density (RBD) and Silt emerged as significant factors driving the variation. Latitude (Lat), altitude (Alt), and mean warmest month temperature (MWMT) were also identified as key influences. HP analysis revealed Silt as the most important predictor (*p* < 0.05). Latent variable analysis indicated descending contribution rates: SPs (31.51%) > GCs (11.52%) > CFs (11.04%) > UVRFs (10.29%). Co-effect analysis highlighted significant coupling effects involving RBD and cation exchange capacity of clay (CECC), as well as organic carbon content (OCC) and UV-B seasonality (UVB2). Path analysis showed SPs as having the strongest influence on leaf thickness (LT), followed by GCs and UVRFs. These findings provide empirical insights into the biogeographical patterns of ITV in *P. chrysandra*, enhance the understanding of plant environmental adaptation mechanisms, and offer a theoretical foundation for studying community assembly and ecosystem function maintenance.

## 1. Introduction

Plant functional traits (PFTs) represent adaptation strategies shaped by long-term environmental pressures, encompassing key biological attributes resulting from the co-evolution of functional modules [1]. These traits, essential for plant growth, development, and reproduction, reflect how plants respond to environmental changes and are optimized through phenotypic integration—the coordinated interactions among traits that influence survival, reproduction, population dynamics and community functions [2,3,4]. This integration is crucial for shaping ecosystem structure, function, and phylogenetic diversity, as it enables plants to adapt holistically to diverse habitats. In the realm of plant-environment interactions, functional traits act as essential conduits for biological information exchange. Leaves hold significant research value due to their extensive surface area and heightened sensitivity to environmental changes [5]. As the primary interface with the environment, they are essential for photosynthesis, carbon assimilation, and transpiration. The evolution of leaf morphology and physiology reflects plants’ strategies for resource acquisition [6]. This combination of traits influences plant niche differentiation and ecosystem energy flux by regulating light-use efficiency, water balance, and stress resistance. Leaf traits not only influence physiological processes at the individual and species levels but also function at larger scales by affecting ecosystem functions and processes, including productivity, biogeochemical cycles, resilience, and hydrological processes [7,8]. Furthermore, the evolutionary trade-off mechanisms of leaf traits provide a critical window for elucidating species adaptive strategies and serve as the cornerstone of species identification and classification [9,10]. In resource-limited environments, plants exhibit a spectrum of adaptive strategies ranging from conservative to acquisitive by modulating traits such as specific leaf area (SLA), stomatal conductance, and chlorophyll content (CHL). This phenotypic plasticity reflects the optimization of photosynthetic resource allocation under natural selection and influences functional diversity gradients within plant communities, thereby contributing significantly to the maintenance of ecosystem services. Currently, integrating functional ecology with evolutionary developmental biology has become a forefront strategy for understanding plant adaptation to environmental changes [5,11]. Trait-based methodologies offer a robust framework for forecasting the effects of climate change on forest ecosystems.

Intraspecific trait variation (ITV), which reflects species’ adaptive responses to environmental filtering and biotic interactions, is critical for elucidating community dynamics and their underlying mechanisms. By linking species’ survival, growth, and reproductive strategies, ITV can reveal species-environment interaction patterns and deepen insights into community structure and ecosystem functioning [12,13,14]. Recent studies have highlighted that ITV is significant, influencing community dynamics and ecosystem stability. For instance, ITV accounts for 25% to 31% of trait variation in plants [13], and in some cases, up to 40% [14]. This variation is crucial for species’ abilities to adapt to new environments or resist environmental changes, with harsher environments amplifying its importance [15,16]. ITV results from both genetic diversity and phenotypic plasticity, providing species with adaptive flexibility. This makes the study of ITV essential for understanding species diversity and predicting how environmental factors shape functional traits.

*Polyspora* Sweet, an evergreen genus, ranks second in species diversity within the Theaceae family, trailing only *Camellia* L., exhibits remarkable ecological resilience and is used widely in landscaping and reforestation. The genus demonstrates remarkable ecological resilience, thriving in rugged mountainous environments with high wind tolerance and efficient regenerative capacity [17]. Consequently, it serves as a keystone pioneer species in reforestation efforts for degraded mountainous regions in southern China. *Polyspora* holds substantial ecological significance, contributing to biodiversity conservation, soil and water retention, watershed management, and urban greening. It also has economic value, with applications in food, medicine, and timber production. Notably, six *Polyspora* species are classified as threatened in both The China Biodiversity Red List-Higher Plants (2020) [18] and The Red List of Theaceae [19], underscoring their vulnerable conservation status. This underscores the urgent necessity for targeted conservation strategies for this genus.

Southwest and South China constitute the primary distribution regions of *Polyspora* species, hosting abundant germplasm resources. Yunnan Province alone harbors three naturally occurring species: *Polyspora speciosa* (Kochs) Bartholo & T. L. Ming, *P. chrysandra* (Cowan) Hu ex Barthol. & Ming, and *P. longicarpa* (Hung T. Chang) C. X. Ye ex Bartholomew & Ming. Previous studies have significantly advanced taxonomy [20], cultivation [21], phytochemistry [22], community ecology [23], and biogeography [24]. These interdisciplinary efforts are crucial for forest resource conservation and the development of specialty tree species. However, due to inadequate basic research and the delayed initiation of development and utilization efforts, only *P. speciosa* and *P. longicarpa* are sporadically used in urban landscaping projects in limited regions. The majority of species remain in their natural habitats, with large-scale applications yet to be realized.

Presently, systematic studies on PFTs of *Polyspora* remain extremely scarce [25], particularly lacking in-depth analysis of the mechanisms linking PFTs to environmental factors and the patterns of ITV. This research gap has directly led to two critical issues: on one hand, it limits the scientific understanding of how *Polyspora* species adapt to complex habitats through PFTs’ adjustments, hindering the elucidation of their ecological adaptation strategies and key limiting factors; on the other hand, it results in a lack of precise support for conservation practices (e.g., delineation of in situ conservation units) and resource development (e.g., selection of superior germplasm) of this genus. Compared with the other two related species, *P. chrysandra*—the most widely distributed and geographically extensive—provides ideal research material to address these issues. Its broad habitat adaptability implies substantial significant ITV in LFTs. Due to the spatial sampling design’s inability to directly disentangle contributions from genetic differentiation versus phenotypic plasticity, we propose that the observed trait variation is predominantly driven by phenotypic plasticity, as corroborated by evidence from analogous studies [6,11]. Accordingly, we selected *P. chrysandra* as the focal study organism and conducted a systematic survey of natural populations across Yunnan Province. Using a spatial gradient sampling approach to investigate trait variation under divergent environmental conditions, we quantified 20 LFTs in mature, undamaged individuals. We gathered data for 33 environmental factors, which were categorized into four groups: geographical conditions (GCs), climatic factors (CFs), soil properties (SPs), and ultraviolet radiation factors (UVRFs). UVRFs primarily affects plant physiological processes through photochemical reactions, with a mechanism of action distinct from other climatic or geographical factors [26]. Thus, this study categorizes it as an independent factor to more precisely elucidate its effects on LFTs. Following multivariate variable screening, Redundancy Analysis (RDA) and hierarchical partitioning (HP) were employed to resolve trait–environment associations and quantify predictor importance. Partial least squares structural equation modeling (PLS-SEM) was further utilized to disentangle direct/indirect pathways mediating environmental effects on LFTs’ variation. We aimed to address the following questions:

(1) What are the patterns of ITV in *P. chrysandra* across different biogeographical regions of Yunnan Province? Given the high environmental heterogeneity in its distribution area, we hypothesize that under the synergistic effects of environmental factors such as GCs, CFs, SPs, and UVRFs, the ITV exhibits a moderate or strong variation level while also demonstrating a broader range of variability (Hypothesis 1).
(2) Which environmental factors drive the variation in LFTs, and what are their relative importance? Previous studies have indicated that ITV is comprehensively regulated by multiple environmental factors [13,15,16]. Therefore, we hypothesize that GCs, CFs, SPs, and UVRFs collectively drive the ITV; among these, owing to the intense ultraviolet radiation (UV) in Yunnan Province, UVRFs likely exert a pronounced effect on the ITV (Hypothesis 2).

## 2. Results

### 2.1. Leaf Functional Traits Characteristics of P. chrysandra

The coefficient of variation (CV) of LFTs in *P. chrysandra* ranged from 7.071% to 25.650% (Table 1, Figure S1). Based on classification criteria for variation degrees from relevant studies [27,28], leaf area (LA), leaf dry weight (LDW), leaf fresh weight (LFW), SLA, and leaf tissue density (LTD) exhibited moderate variation (20% < CV ≤ 50%) with CV values of 25.650%, 23.544%, 22.528%, 22.292%, and 21.941%, respectively; the remaining traits showed weak variation (CV ≤ 20%). Among these, leaf water concentration (LWC, CV = 7.071%) and leaf dry matter content (LDMC, CV = 9.255%) had the lowest CV, indicating stable performance within the population. Phenotypic plasticity index (PI) analysis revealed trait PI values ranging from 0.249 to 0.710 (Figure S1): LA (PI = 0.710), SLA (PI = 0.612), and LTD (PI = 0.607) displayed high plasticity (PI > 0.6), while leaf shape factor (LSF, PI = 0.249), LWC (PI = 0.263), and leaf weight ratio (LWR, PI = 0.271) were conservative (PI < 0.3).

Further analysis showed a significant positive correlation between CV and PI (r = 0.974, *p* < 0.01), indicating an inherent link between trait variation magnitude and environmental responsiveness. Beyond variation and plasticity, trait distribution characteristics also revealed population adaptive strategies. Skewness and kurtosis analysis showed that LDW exhibited a significantly right-skewed distribution (Skewness = 1.335) and leptokurtic distribution (Kurtosis = 2.365), indicating most individuals had low LDW values with a few extreme high values—this is associated with niche differentiation driven by resource heterogeneity, where low-LDW individuals adopt rapid resource acquisition to adapt to poor habitats, while high-LDW individuals enhance structural defense to exploit local high-resource microhabitats. CHL showed a slightly left-skewed distribution (Skewness = −0.279), reflecting population preference for moderate-to-high light environments, with the left-skewed tail potentially representing adaptive phenotypes for shaded edge habitats. These non-normal distribution characteristics indicate that *P. chrysandra* maintains population stability in heterogeneous environments through a “risk-spreading strategy” of convergent adaptation in most individuals and specialized adaptation in a few.

### 2.2. Variable Screening Results

#### 2.2.1. Screening Results of Response Variables

**Figure 1:**
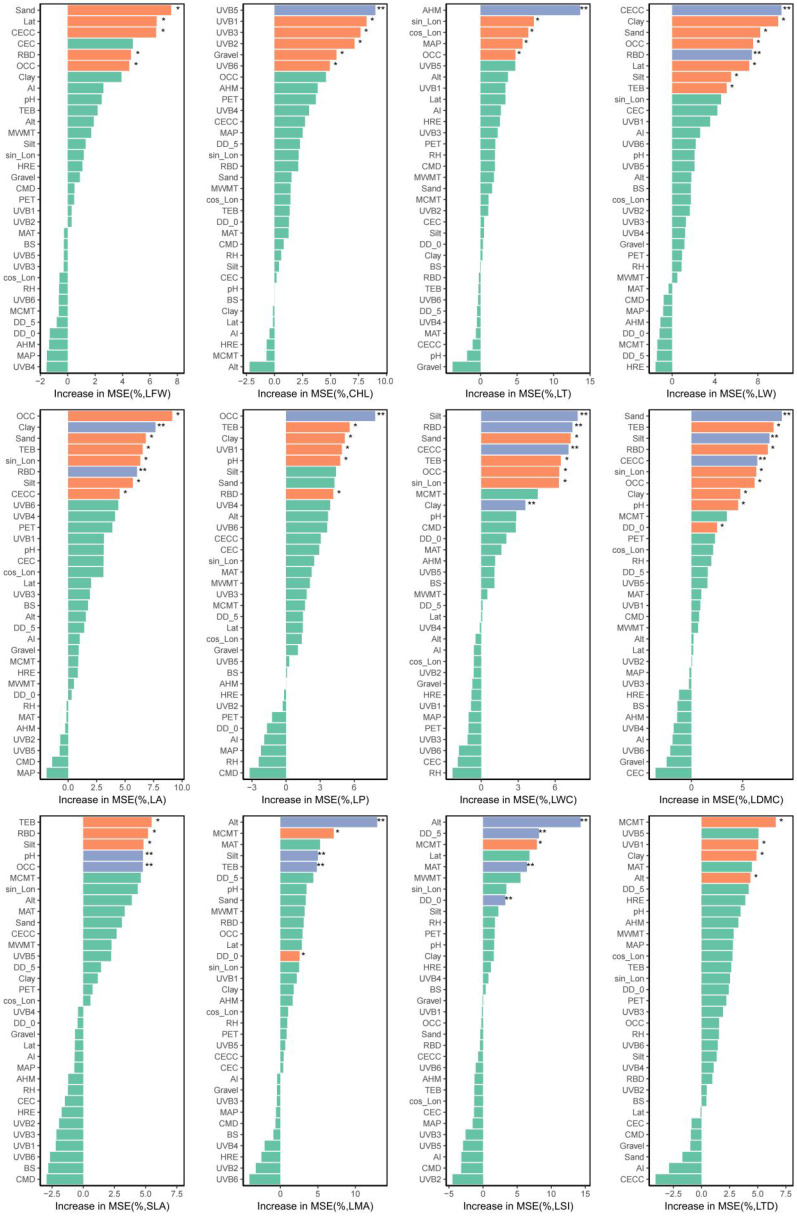
Top 12 LFTs were identified using the RF algorithm. The ranking of traits was determined by the predictive importance of environmental variables, quantified as IncMSE when each variable was permuted. Significance levels: ** *p* < 0.01 (blue), * *p* < 0.05 (orange), and non-significant (green), as indicated in the figure.

A total of 33 environmental factors were categorized into four distinct groups: GCs, CFs, SPs, and UVRFs, and were subsequently employed as predictive variables. The Random forest (RF) algorithm was implemented to rank and prioritize 20 response variables, utilizing the percentage increase in Mean Squared Error (IncMSE, %) as the evaluation metric for variable importance quantification. Variable importance was determined by the magnitude of IncMSE values, with results systematically presenting the importance scores and statistical significance levels for these factors (Table S1). Notably, Aridity index (AI), Climatic moisture deficit (CMD), Hargreaves reference evapotranspiration (HRE), Mean warmest month temperature (MWMT), Relative humidity (RH), Potential evapotranspiration (PET), Base Saturation (BS), Cation Exchange Capacity (CEC), and mean UV-B of the lowest month (UVB4) demonstrated zero significance frequency in IncMSE, indicating negligible explanatory capacity and justifying their exclusion from subsequent analyses. Conversely, Clay, Organic Carbon Content (OCC), Reference Bulk Density (RBD), and Total Exchangeable Bases (TEB) each exhibited a significance frequency of 7, while Silt registered 6 occurrences. Cation Exchange Capacity of Clay (CECC) and Sand both displayed 5 occurrences, with altitude (Alt) and sin_Lon (sine-transformed longitude) scoring 4 each. Latitude (Lat), Degree-days below 0 °C (DD_0), Mean annual temperature (MAT), Mean coldest month temperature (MCMT), pH, and Annual mean UV-B (UVB1) each recorded 3 occurrences, whereas cos_Lon (cosine-transformed longitude) and Annual heat: Moisture Index (AHM) showed 2 occurrences. The remaining factors each exhibited a single occurrence. Among the 20 LFTs ranked by IncMSE significance, the hierarchy was as follows: LDMC (10) > LA (8) = leaf width (LW) (8) = LWC (8) > CHL (6) = leaf perimeter (LP) (6) > leaf mass per unit area (LMA) (5) = leaf succulence index (LSI) (5) = SLA (5) > LFW (4) = leaf thickness (LT) (4) = LTD (4) > LWR (3) > leaf length (LL) (2) = petiole length (PL) (2) > LSF (1) = petiole diameter (PD) (1) > LDW (0) = leaf serration density (LSD) (0) = leaf serration number (LSN) (0). Based on these rankings, the top 12 traits—LDMC, LA, LW, LWC, CHL, LP, LMA, LSI, SLA, LFW, LT, and LTD—were selected for in-depth investigation (Figure 1).

#### 2.2.2. Screening Results of Explanatory Variables

The 12 LFTs—LDMC, LA, LW, LWC, CHL, LP, LMA, LSI, SLA, LFW, LT, and LTD—were evaluated using the RF algorithm to quantify the relative importance of each environmental predictor. Variable retention followed a strict criterion: only predictors with importance scores exceeding the maximum value of shadow features (permuted replicates) were retained for subsequent analysis, while non-significant predictors were systematically excluded.

Through the Boruta feature selection process, all GC indicators received “Confirmed” status. Within the UVRF category, UVB1, UV-B seasonality (UVB2), mean UV-B of the peak month (UVB3), total monthly mean UV-B during the peak quarter (UVB5), and total monthly mean UV-B during the lowest quarter (UVB6) were “Confirmed”. In the CF group, MCMT, DD_5, AHM, MWMT, MAP, and DD_0 attained “Confirmed” status. Among SP indicators, OCC, TEB, RBD, Silt, Sand, CECC, Clay, and pH were “Confirmed”, whereas remaining variables were “Rejected”. Ultimately, 23 environment variables with “Confirmed” status were selected as predictive variables for downstream analyses (Table S2).

#### 2.2.3. RDA Analysis of Leaf Functional Traits and Environmental Factors and Interpretation of Relative Importance

**Figure 2:**
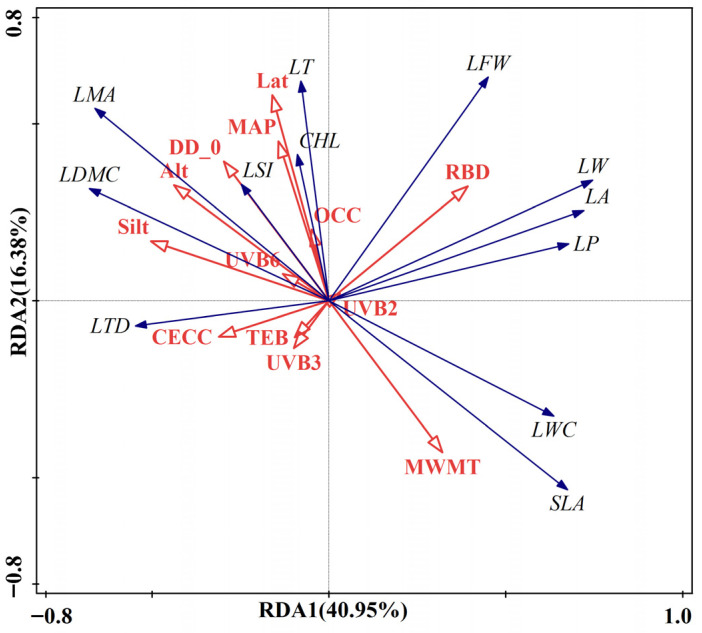
RDA triplot illustrating the relationships between LFTs (blue arrows) and environmental factors (red arrows). Percentages on axes indicate explained variation (Total explained variation: 64.70%, adjusted R^2^ = 31.9%; *p* = 0.026, permutation test with 999 iterations).

Detrended Correspondence Analysis (DCA) ordination axis exhibited a maximum gradient value of 0.219, which is below the threshold of 3, thus justifying the selection of RDA for evaluating the environmental drivers of LFTs. The explanatory variables included 13 environmental factors screened via the Boruta algorithm and VIF analysis (VIF < 10). These factors encompassed Lat, Alt, MWMT, MAP, DD_0, UVB2, UVB3, UVB6, Silt, RBD, OCC, CECC, and TEB. The RDA results presented in Figure 2 demonstrate that RDA1 and RDA2 jointly explained 57.33% of the variance in LFTs of *P. chrysandra*. In the RDA ordination diagram, the arrow length corresponding to each environmental factor denotes the magnitude of its eigenvector loading, reflecting its relative explanatory power for LFT variation. The acute angle between two arrows indicates positive correlation, obtuse angles (90°~180°) denote negative correlation, while orthogonal angles (90°) suggest statistical independence between variables [29]. Figure 2 reveals that Lat, Alt, RBD, Silt, and MWMT emerge as the dominant environmental drivers of LFTs. RBD exhibits positive associations with multiple LFTs, including LFW, LW, LA, LP, LWC, SLA, LT, and CHL. Lat and Alt primarily exert positive influences on LT, CHL, LSI, LMA, and LDMC, whereas MWMT shows negative effects on these traits but positive effects on LWC, SLA, LP, LA, and LW. Conversely, Silt predominantly demonstrates negative relationships with LW, LA, LP, LWC, and SLA. These findings collectively indicate that SPs (RBD, Silt) significantly modulate LFTs, with GCs (Lat, Alt) and CFs (MWMT) also serving as critical determinants of LFT variation. These variables collectively constitute the primary drivers of functional trait divergence in *P. chrysandra*.

**Figure 3:**
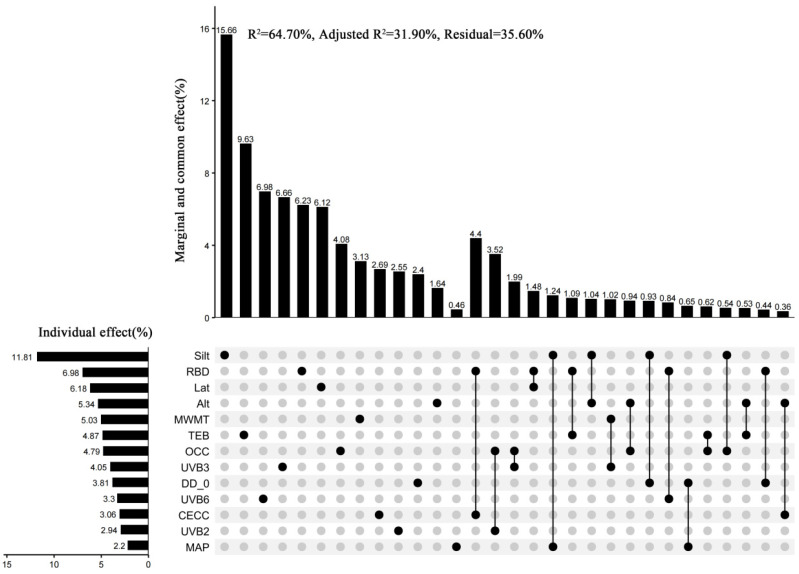
HP and VP UpSet plot illustrating the relative importance of environmental factors on LFTs of *P. chrysandra*. In the matrix plot on the right, each row corresponds to an environmental factor. For each column, isolated black dots denote the marginal effects of individual environmental factors, while connecting lines between multiple points signify the joint effects among these factors. The percentage of variance explained by each component (derived from VP) is displayed in the bar chart above. The bar chart on the left represents the individual effects of each environmental factor (from HP), with values equating to the marginal effect of the factor plus the equally apportioned joint effects with other environmental factors.

HP and variation partitioning (VP) analyses (Figure 3) revealed that Silt emerged as the dominant environmental driver of LFT variation, contributing 11.81% to the explanatory power—the highest relative importance among all individual factors (*p* < 0.05). RBD (6.98%), Lat (6.18%), Alt (5.34%), MWMT (5.03%), and TEB (4.87%) constituted secondary contributors. When aggregated into latent constructs, the explanatory power shifted to 11.52% (GCs), 11.04% (CFs), 10.29% (UVRFs), and 31.51% (SPs). For interactive effects, the most influential environmental factor pairs influencing *P. chrysandra* LFTs were RBD + CECC (4.40%) and OCC + UVB2 (3.52%). Other factor combinations exhibited comparable explanatory power, with joint contribution thresholds ranging from 0.36% to 1.99%.

### 2.3. Results of CCA and the Optimal PLS-SEM Model

**Figure 4:**
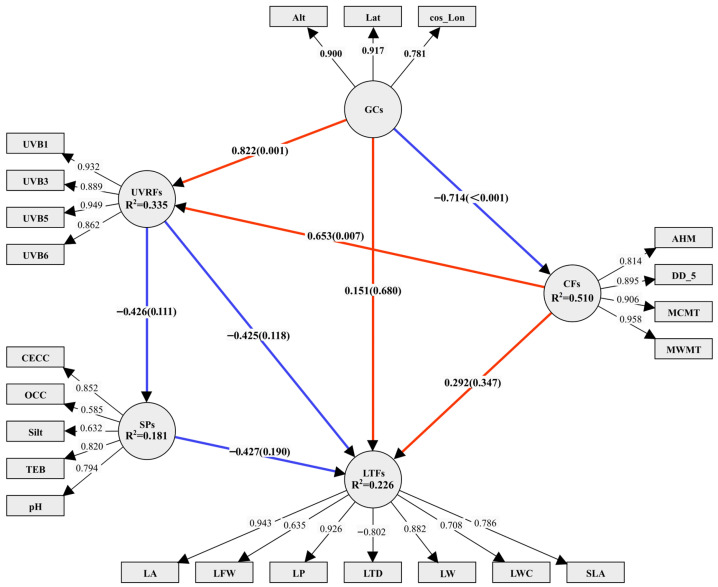
Optimal PLS-SEM linking environmental factors and LFTs. Note: Circles represent latent variables, and single-headed arrows indicate the path relationships between these variables. The red arrow in the figure indicates a positive impact, while the blue arrow indicates a negative impact. The values outside the parentheses represent path coefficients, and the values inside the parentheses indicate significance levels (*p* < 0.05).

The results of Canonical Correlation Analysis (CCA) analysis (Table 2) show that the Rc values between the latent variable groups range from 0.627 to 0.999, with 6 groups having Rc > 0.8, indicating a strong overall association. However, only 6 paths simultaneously meet the criteria of Rc ≥ 0.6 and *p* < 0.05: GCs-CFs, GCs-LFTs, SPs- LFTs, GCs-UVRFs, CFs-UVRFs, and UVRFs-SPs. These six pairs of latent variable groups were initially included in the structural equation model paths, while SPs-LFTs associations were simultaneously integrated. Conversely, GCs-SPs (*p* > 0.05) and CFs-SPs (*p* > 0.05) pathways were excluded due to non-significance. Notably, despite lacking statistical significance, CFs-LFTs (Rc = 0.905) and UVRFs-LFTs (Rc = 0.999) exhibited strong canonical correlations. These pathways were provisionally retained in the initial PLS-SEM to ensure exploratory rigor in latent variable relationship mapping. Model validation and refinement subsequently evaluated the structural impact of these pathways on overall model stability. Pathways were retained only if their exclusion significantly compromised model explanatory power. The baseline model was constructed using SmartPLS software (version 4.1.0.3), employing the “PLS-SEM algorithm”, “Bootstrapping”, and “Blindfolding” procedures for parameter estimation and cross-validation. Iterative model validation was performed to achieve optimal model fit, adhering to all established validation criteria (Figure 4). It is noteworthy that this study observed the coexistence of high Rc values with non-significant paths, which confirms the independence of effect size and significance in CCA. Sample size limitations and residual collinearity may reduce the significance of weak effect paths. Future studies could further validate these findings by increasing the sample size or using Partial Least Squares Canonical Correlation Analysis (PLS-CCA).

Table 3 presents the Composite reliability (CR) values for latent variables in the optimized PLS-SEM model, ranging from 0.790 to 0.989, indicating acceptable internal consistency. Concurrently, Cronbach’s α coefficients (0.731~0.954) exceeded the 0.7 threshold, confirming adequate scale reliability. The average variance extracted (AVE) values (0.554~0.881) surpassed the 0.5 discriminant validity criterion, demonstrating satisfactory convergent validity and construct clarity. Discriminant validity was verified through two complementary approaches: (1) Cross-loading analysis (Table S3) confirmed that item loadings on their respective constructs exceeded cross-loadings on other latent variables; (2) Heterotrait–Monotrait (HTMT) ratio analysis (Table S4) showed HTMT values (0.336~0.724) below the 0.85 threshold, reinforcing construct distinctiveness. Notably, the first five columns of Table S4 validate that all AVE values meet the Fornell–Larcker criterion, while the final five columns confirm HTMT-based discriminant validity. Formative indicator multicollinearity was assessed via VIF, with all values below 3.3, satisfying diagnostic criteria. Predictive relevance was confirmed by Q^2^ values > 0 (Stone-Geisser criterion), and model goodness-of-fit (GoF) reached 0.481—exceeding the 0.36 threshold—indicating strong explanatory power and theoretical coherence.

### 2.4. Assessment of Influencing Factors of Leaf Functional Traits

**Figure 5:**
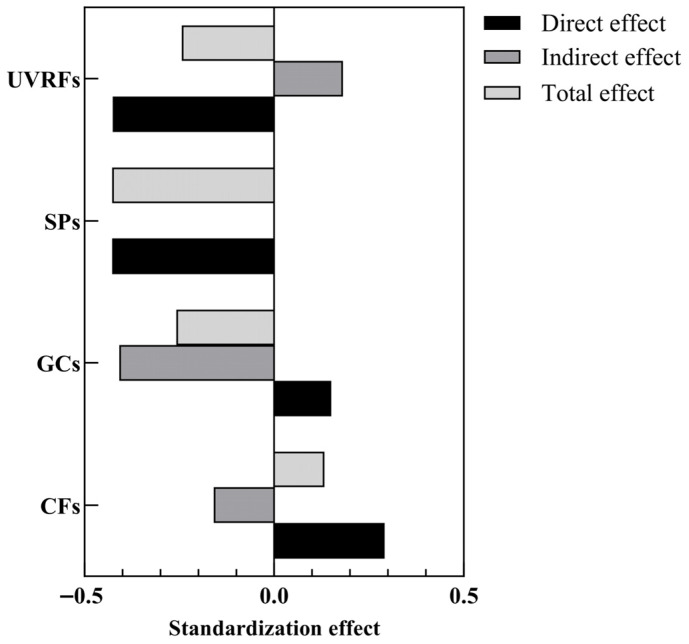
Standardized effects of environmental factors on LFTs of *P. chrysandra*.

Executing “PLS Algorithm” in SmartPLS yields R^2^ values ranging from 0.181 to 0.510, exceeding the 0.1 threshold and indicating acceptable explanatory power for the structural model [30]. The optimized SEM framework (Figure 4) revealed that GCs, CFs, SPs, and UVRFs jointly explained 22.60% of the variance in LFTs. Path analysis demonstrated that CFs, GCs, and UVRFs exerted both direct and mediated influences on LFTs. Specifically, CFs exhibited a total effect of 0.133 (comprising 0.292 direct effect and −0.159 indirect effect), while GCs showed a total effect of −0.257 (combining 0.151 direct effect and −0.408 indirect effect). UVRFs demonstrated a total effect of −0.243 (with −0.425 direct effect and 0.182 indirect effect), whereas SPs manifested a solely direct effect of −0.427. Collectively, SPs emerged as the dominant predictor of LFT variation (total effect: −0.427), followed by GCs (total effect: −0.257) and UVRFs (total effect: −0.243), with CFs exerting the weakest influence (total effect: 0.133) (Figure 5).

“PLS Algorithm” and “bootstrapping” analyses revealed that the measurement model’s path relationships were statistically significant (*p* < 0.05), with minimal non-significant exceptions, underscoring the model’s robust explanatory capacity. Specifically, the loading coefficients for LA (0.943), LP (0.926), LTD (−0.802), LW (0.882), and SLA (0.786) achieved extreme statistical significance (*p* < 0.001), while LFW (0.635) and LWC (0.708) attained conventional significance (*p* < 0.05). Alt (0.900), Lat (0.917), and cos_Lon (0.781) also exhibited paramount significance (*p* < 0.001). Similarly, UVB1 (0.932), UVB3 (0.889), UVB5 (0.949), and UVB6 (0.862) reached extreme significance (*p* < 0.001), with UVB1 and UVB5 demonstrating comparable magnitude of influence. The loading coefficients for AHM (0.814), DD_5 (0.985), MCMT (0.906), and MWMT (0.958) were highly significant (*p* < 0.001), with DD_5, MCMT, and MWMT exhibiting equivalent explanatory power. CECC (0.852), Silt (0.632), TEB (0.820), and pH (0.794) achieved moderate significance (*p* < 0.01), while OCC showed no statistical significance (*p* > 0.05). Collectively, these results identify Lat, UVB1, UVB5, CECC, TEB, DD_5, MCMT, and MWMT as the primary determinants of functional trait variation in *P. chrysandra* leaves.

## 3. Discussion

### 3.1. Variation Characteristics of Leaf Functional Traits of P. chrysandra

The variation patterns and broad variability in LFTs of *P. chrysandra* reveal its multi-level adaptation mechanisms to environmental heterogeneity. Specifically, traits closely associated with resource acquisition and utilization—including LA, SLA, LTD, LDW, LFW—display moderate variation levels, whereas other traits show lower variability. These results partially support our first hypothesis. LA and SLA showed significant coordinated variation, likely driven by soil nutrient heterogeneity and precipitation gradients [31]. This pattern aligns with *Distylium chinense* (Franch. ex Hemsl.) Diels and *Phoebe bournei* (Hemsl.) Yen C. Yang in different habitats [32], indicating a resource-driven strategy where leaf expansion enhances light capture while reducing mass-based photosynthetic costs—a hallmark of pioneer species [33]. Such traits confer adaptive advantages in early secondary succession or post-disturbance environments. Epigenetic regulation and flavonoid accumulation may underpin this plasticity [34], with seasonal UV fluctuations inducing cuticle thickening and flavonoid synthesis to mitigate UV damage [35]. This metabolic-morphological synergy forms the core adaptive mechanism for responding to canopy light dynamics.

The strong PI-CV correlation (r = 0.974) in LFTs suggests high-variability traits exhibit greater environmental responsiveness. CV could complement existing metrics in evaluating trait plasticity for predicting ecological adaptation, especially in assessing niche occupancy potential in pioneer species. Low-variability traits (e.g., LWC, LDMC) reflect conservative strategies, likely developed under periodic drought stress in southwestern mountainous regions. Stable turgor maintenance and rigid cell walls ensure water use efficiency while reducing wilting risks, demonstrating functional convergence with drought-resistant *Camellia* species, such as *Camellia oleifera* Abel and *Camellia petelotii* (Merr.) Sealy [36,37].

PI values revealed plastic differentiation: high-PI traits (LA, SLA, LTD; PI > 0.6) adopt a “fast-investment” strategy typical of pioneer species [38], enhancing light acquisition and biomass accumulation through coordinated LA-SLA regulation. This strategy is typical of pioneer species, suggesting that *P. chrysandra* fits this category and can swiftly occupy ecological niches following disturbances. Low-PI traits (LSF, LWC, LWR; PI < 0.3) exhibit genetic conservatism, stabilizing physiological processes by limiting trait fluctuations [39]. This equilibrium between plasticity and conservatism may be the fundamental mechanism allowing *P. chrysandra* to optimize resource-use efficiency while mitigating survival risks across habitats.

The CV-PI synergy observed in this study provides novel insights into *P. chrysandra*’s ecological amplitude expansion mechanism, where high-variability populations demonstrate superior adaptation potential. This finding advances plant distribution prediction under climate change. While both indicators characterize trait features, single-metric limitations [40,41] necessitate combined CV-PI analysis to capture multi-dimensional trait variability. Future cross-scale studies (individual–population– community) should validate CV-PI correlation universality.

Distribution skewness and kurtosis offer indirect insights into environmental selection pressures. The right-skewed, leptokurtic distribution of LDW (Skewness = 1.335) likely indicates a directional biomass allocation towards cell wall components like lignin under drought stress [42]. This asymmetric adaptation enhances leaf mechanical strength, bolstering resistance to wilting. Conversely, the slight left-skewed distribution of CHL (Skewness = −0.279) may be associated with the light adaptation strategy in canopy vertical stratification. In high-light environments, plants may downregulate chlorophyll synthesis to avoid photoinhibition damage [43]. This study reveals that *P. chrysandra* adapts to diverse resource environments through a dual strategy: “highly plastic traits enabling immediate environmental responses and conservative traits ensuring physiological stability”. This pattern may be common among shrubs that possess both pioneer traits and stable habitat requirements. This trait interplay provides new perspectives on plant functional trade-offs.

### 3.2. Effects of Soil Properties on Leaf Functional Traits of P. chrysandra

SPs and PLTs exhibit interdependent regulation that shapes ecological adaptation. SPs directly modulate resource acquisition and physiological processes, while plants adjust LFTs to optimize fitness under soil constraints. This dynamic influences both individual performance and ecosystem-level functionality. According to the results of RF, physical (Clay, Sand, Silt, RBD, Gravel) and chemical (OCC, TEB, CECC, pH) properties were included in the PLS-SEM model. These indicators strongly explain LFTs and drive trait variation. SPs interact to influence plant growth and function. Key environmental factors like inorganic nutrients, organic matter, pH, and moisture affect plant growth and function. These factors interact complexly. For example, organic matter improves soil structure, water retention, and nutrient retention, indirectly boosting water use efficiency and nutrient absorption [44].

RBD regulates plant leaf morphology and stoichiometry by affecting root growth, water infiltration, and nutrient availability. For example, LDMC and LT in *Pinus sylvestris* var. *mongholica* Litv. increase with higher RBD [45]. Increased Gravel content generally leads to higher soil porosity and reduced water retention, prompting plants to reduce SLA or increase LT to minimize transpiration losses [46]. Soils with high Clay and Silt retain water and nutrients but have poor aeration. In these soils, plants tend to increase SLA to improve photosynthetic efficiency. The Clay positively correlated with the relative chlorophyll content (SPAD) of *Pinus massoniana* Lamb., indicating that Clay may enhance photosynthesis by improving nitrogen uptake [46]. Higher Gravel restricts root expansion, leading plants to allocate more resources to above-ground parts to compensate for reduced root absorption efficiency. This results in higher SLA and lower LDMC, enabling rapid capture of light resources [47,48]. However, when Gravel and Clay work in synergy, Clay filling gaps in Gravel may enhance soil water retention, indirectly alleviating leaf water stress.

Soil chemical indicators (OCC, TEB, CECC, pH) affect LFTs by regulating nutrient availability, root uptake, and microbial activity. OCC is positively correlated with OCC leaf total nitrogen (TN) in *Quercus glauca* Thunb. [48] and may increase the leaf carbon–nitrogen ratio when binding to Clay, delaying mineralization [49]. TEB increases leaf calcium and magnesium content by increasing BS. Base cations may also affect leaf transpiration by adjusting stomatal conductance, improving water potential, and reducing LDMC to optimize photosynthesis. CECC helps maintain leaf nutrient balance by adsorbing cations, reducing nutrient leaching, and lowering leaf nitrogen and phosphorus content. pH influences LFTs, by affecting stoichiometry, microbial-leaf interactions and defense traits [45,50,51].

RDA results showed that Silt and RBD had significant positive correlations with most LFTs. HP analysis further revealed that Silt and RBD explained 11.81% and 6.98% of the variation, respectively, higher than other SPs. This highlights their dominant independent effects on the variation of LFTs. The total explanatory contribution of SPs was 31.51%, emphasizing the substrate environment’s role in leaf resource allocation. PLS-SEM path analysis demonstrated that CECC (*p* < 0.01), Silt (*p* < 0.05), TEB (*p* < 0.01), and pH (*p* < 0.01) as key drivers of LFTs’ variation.

The total effect of SPs on LFTs was −0.427, higher than other environmental factors, indicating their primary role in shaping LFTs. This negative effect suggests that SPs inhibit LFTs, likely through soil factors like nutrient availability, pH, and moisture, which limit traits such as photosynthetic efficiency, rate, and LA. Excessive salinity or nutrient deficiency, for example, can restrict plant growth and affect LFTs [29]. These findings align with studies on karst forest succession [52] and coastal vegetation [53], which emphasized soil’s dominant role in LFTs’ differentiation, implying a universal SPs regulatory mechanism on LFTs across different ecosystems.

### 3.3. Effects of Ultraviolet Radiation Factors on Leaf Functional Traits of P. chrysandra

This study revealed that, except for UVB4, the other five UVRFs all play significant regulatory roles in LFTs of *P. chrysandra*, which is highly consistent with the natural geographical conditions of strong UV in high-altitude regions of Yunnan Province. UV affects LFTs through several mechanisms, notably inhibiting chlorophyll synthesis, especially at high altitudes. For example, studies on *Fraxinus mandshurica* Rupr. revealed that UV intensity and altitude reduce chlorophyll content, while carotenoids and flavonoids increase to protect the photosystem from photoinhibition [54].

Additionally, UV not only directly interferes with chloroplast function by accelerating structural damage but also activates the chlorophyll degradation pathway, creating a dual inhibitory effect [55]. This response reflects the plant’s strategy: reducing CHL to minimize UV absorption risks while redirecting resources to secondary metabolites, forming a multi-layered photoprotective system. This adaptation helps plants thrive in high-radiation environments. In this study, multiple UV radiation indicators were significantly linked to CHL, confirming the central role in regulating LFTs.

Furthermore, existing studies demonstrated UV affects LFTs across morphology, physiology, and stoichiometry, involving complex physiological and molecular mechanisms like signal pathways, gene expression, secondary metabolism, DNA repair, and antioxidants. However, these studies only confirmed UV’s impact on LP and LTD, without exploring physiological, or molecular mechanisms. Future research should focus on these underlying processes.

RDA results demonstrated positive correlations between UVB2 and LFW, LW, LA, LP, LWC, and SLA. UVB3 correlated with LTD and LDMC, while UVB6 linked to LDMC, LMA, LTD, LSI, and LT. HP analysis revealed independent explanatory variances of 2.94% for UVB2, 4.05% for UVB3, and 3.30% for UVB6, totaling 10.29%. This cumulative variance was lower than other factors, suggesting a subsidiary role in regulating LFTs. Notably, discrepancies emerged between the RDA and HP results regarding the ecological weighting of UVRFs. RDA captures the aggregate explanatory power of environmental variables (including multicollinear components), potentially obscuring the subsidiary influence of UVRFs due to covariance with other factors. Conversely, HP isolates the unique explanatory capacity of each variable by controlling for confounding effects, thereby uncovering the distinct contribution of UVRFs (10.29%)—a specialized ecological function independent of SPs or GCs. PLS-SEM path analysis identified UVB1, UVB3, UVB5, and UVB6 as principal drivers of LFT variation (*p* < 0.001), with UVRFs exerting a total effect of −0.243 on LFTs. This suggests UV radiation may reduce photosynthetic capacity, antioxidant defenses, and DNA integrity, influencing leaf trait expression [56].

### 3.4. Effects of Geographical Conditions on Leaf Functional Traits of P. chrysandra

GCs drive adaptive divergence in PFTs by regulating light, heat, water and soil. Altitude is the main geographic factor influencing LFTs. SLA decreases with increasing altitude, which is attributed to the tendency of plants to reduce the light capture area and increase the chloroplast density to avoid photoinhibition under low temperatures and high radiation. High-altitude plants usually have larger LT and higher LDMC due to cell wall thickening and mechanical reinforcement, which helps to resist wind and frost [57,58]. LMA and LTD also increase with altitude, reflecting stress from low temperature, strong radiation and poor soil [6]. This study found altitude additionally affects the LSI of *P. chrysandra*, suggesting LSI is shaped by multiple factors such as temperature, water and soil. Latitude and longitude mainly affect LFTs indirectly through environmental changes. Higher latitudes reduce temperature and alter light conditions, affecting photosynthesis, growth, leaf morphology, and stoichiometry. Plants at high latitude tend to have higher cold tolerance and lower photosynthetic rates. Longitude affects precipitation and humidity, influencing water use efficiency, leaf morphology, and physiology. In this study, longitude primarily influenced LDMC, LA, LWC, and LT, while latitude mainly affected LW and LFW. These findings are broadly consistent with previous research [6]. However, certain distinct characteristics and differences were observed. Specifically, the influence of longitude was greater than that of latitude, with many significant indicators. The primary reason for this may be that the response of LFTs of *P. chrysandra* to soil characteristics and precipitation is more sensitive than its response to temperature and illumination. Alternatively, the influence of latitude and longitude on LFTs may not exist independently, and the observed variation may not solely result from the independent effects of latitude and longitude, soil, and CFs, but may also be driven by their interactive effects. Moreover, the wider longitudinal span of the sampling sites selected in this study, compared to the latitudinal span, may also contribute to the stronger influence of longitude on LFTs.

In addition, Topography also shapes LFTs by altering local light, temperature, water, and nutrients. Slope aspect increases LDMC under high sunlight and poor soils, while slope steepness affects soil water retention, nutrient availability, and microclimate, influencing leaf morphology and elemental composition. These topography-driven variations constrains adaptive evolution by altering SPs such as pH, organic content, and mineral nutrients. Our analysis confirmed significant effects of Lat., Lon., and Alt. on 12 leaf traits, without considering terrain and slope variables. Future studies should explore the combined effects of multi-scale factors on leaf trait adaptation.

RDA results indicated that GCs (Alt., Lat.) positively correlate with LDMC, LMA, LT, LSI, and LW, suggesting that high-altitude and high-latitude environments favor conservative evolutionary strategies. These strategies promote thicker leaves and SLA due to stress from low temperatures and increased radiation [6]. HP analysis further reveals that altitude and latitude contribute 5.34% and 6.18% of leaf trait variation, respectively, with a total of 11.52%, second only to soil attributes. This underscores their significant role in the multifactorial regulation of LFTs, highlighting the influence of geographical differentiation on LES via photothermal gradients. PLS-SEM identified altitude, latitude, and cosine longitude as key drivers (*p* < 0.001). Notably, the total effect value of GCs on LFTs was −0.257, indicating that geographical conditions (e.g., altitude, topography) exert a negative effect on LFTs. This potentially reflects that resource limitation induced by increasing geographical gradients (e.g., nitrogen mineralization constrained by low temperatures at high altitudes) forces plants to adopt trait trade-off strategies: specifically, reducing the turnover rate of photosynthetic products (decreased SLA) in exchange for enhanced structural defense capacity (increased LDMC) [6,59].

### 3.5. Effects of Climate Factors on Leaf Functional Traits of P. chrysandra

CFs such as temperature, precipitation, and light directly influence leaf morphology and physiology. High temperatures typically cause leaves to grow larger and thinner, while low-temperature conditions result in smaller and thicker leaves. These changes help plants survive and grow in different climates. Elevated temperatures can lower LNC and LPC and reduce photosynthesis rates, though some studies suggested increased leaf carbon content with higher temperatures, indicating species variability [60]. Precipitation is a critical factor influencing plant distribution. Extreme precipitation can alter LNC and LPC, affecting photosynthesis and growth. Previous studies have found that in harsh hydrothermal environments with insufficient nutrient supply for plant growth, SLA, LWC, and LA are significantly reduced, while stress resistance is increased [60]. Light intensity exerts dual regulatory effects. High irradiance reduces leaf size/longevity but promotes LA expansion for light capture optimization, though excessive light induces photoinhibition via chloroplast damage. Low-light environments trigger structural reinforcement (↑LT, ↓SLA). Light regimes also modulate CHL dynamics: mild water deficit synergistically elevates CHL content, improving stress resilience while balancing light harvesting and carbon assimilation [61]. These adaptive responses reflect functional trade-offs between resource acquisition efficiency and environmental stress protection.

This study identified climatic factors such as DD_0, MAT, MCMT, AHM, DD_5, and MAP as primary influences on LSI, LMA, LDMC, LT, and LTD, aligning with previous research [6,24,62]. Among these, meteorological factors most closely relate to LSI due to its association with leaf traits such as LDMC, SLA, LT, and LTD, which are regulated by climate. However, this study focuses on the morphological characteristics of *P. chrysandra* leaves, excluding physiological traits due to variability in environmental factors. Future studies should incorporate common garden experiments for further validation.

RDA analysis revealed that MWMT is positively associated with SLA, LWC, LP, and LA, indicating that high temperatures favor traits that enhance light-energy capture. This supports the global LES theory, which suggests that higher temperatures favor resource-acquisition [63]. In contrast, MAP and DD_0 correlated with LMA, LDMC, LT, LSI, and LTD, indicating plant adaptations to drought stress and cold conditions, resulting in conservative economic strategies. This aligns with the “water–low temperature coupling” observed in *Monimopetalum chinense* Rehder [64]. HP disclosed that the individual explanatory rates of MWMT, MAP, and DD_0 were 5.03%, 2.20%, and 3.81%, respectively, totaling 11.04%. Although slightly higher than UVRFs (10.29%), these rates were notably lower than SPs (31.51%), suggesting that climatic influences LFTs indirectly, through factors like temperature-soil interactions and topography [31,64].

PLS-SEM path analysis revealed that AHM, DD_5, MCMT, and MWMT significant drive LFTs’ variation (*p* < 0.001). The total effect of CFs on LFTs was 0.133, indicating an indirect influence through variables like soil, topography, and microbial activity. For instance, temperature affects leaf traits via soil nutrient cycling, while precipitation is influenced by topography [65]. The effects of climate may across ecosystems and plant species, reflecting geographic and temporal differences.

The above findings support our second hypothesis that ITV is comprehensively regulated by multiple environmental factors, with UVRFs exerting a significant influence on ITV. Compared with CFs, UVRFs, and GCs, SPs play a more central role in regulating LFTs. The underlying mechanisms can be explained by two aspects: direct driving at the local scale and synergistic effects of multiple factors. At the local scale, SPs act as direct regulators of plant resource acquisition, shaping LFTs by influencing physiological processes such as photosynthetic enzyme activity and nutrient allocation ratios. In contrast, CFs, UVRFs, and GCs show low spatial variation across sampling sites, and their impacts on LFTs may be masked by soil heterogeneity or indirectly transmitted through cascading pathways. Furthermore, the ecological effects of CFs, UVRFs, and GCs exhibit significant scale dependence: at the regional/global scale, climate and UV dominate the macro-scale patterns of LFTs, while GCs indirectly affect trait distribution by altering hydrothermal combinations. However, at the local scale, the spatial variation of these factors is insufficient to independently drive LFT differentiation, and with most effects being transmitted through SPs. Therefore, the exclusion of some CFs, UVRFs, and GCs in this study is not due to their lack of ecological significance, but rather to their weak local variation and indirect action primarily through soil, thereby playing a subordinate role in direct effect analysis.

### 3.6. Potential Limitations of the Study

In this study, We utilized a combination of PLS-SEM, RDA, and HP to decode the drivers of LFTs’ variation attributable to environmental factors. These methods complemented each other in causal pathway construction, variance quantification, and importance ranking. However, the combined approach has some limitations: PLS-SEM’s theoretical dependence may overlook biological interactions and latent variables, and its linear path assumption cannot capture nonlinear relationships. RDA, based on linear correlation, cannot reflect nonlinear driving modes and may overestimate environmental factors due to covariance. HP, using the “rdacca.hp” package, improves accuracy by evenly distributing shared explanatory rates but is computationally inefficient for high-dimensional variables and relies on linear assumptions, limiting its ability to capture nonlinear interactions. Additionally, it struggles with categorical variables and may become unstable due to extreme covariance, leading to ambiguous biological significance.

## 4. Materials and Methods

### 4.1. Study Area

Yunnan Province, located in the core ecological barrier region of southwest China, features a unique geographical location, a complex geological structure, and diverse climatic conditions. Renowned as the “Kingdom of Plants”, it is one of the global biodiversity hotspots. Situated at the junction of tropical Southeast Asia and subtropical-temperate East Asia and the Himalayas, it serves as a natural “Garden of Eden” for the origin, evolution, and proliferation of life, occupying an irreplaceable position in global biodiversity conservation. The terrain slopes from northwest to southeast, with an elevation span of 6663.6 m. The extreme altitude difference is formed between Kawagebo Peak of Meili Snow Mountain (6740 m) and the confluence of Nanxi River and Honghe River in Hekou County (76.4 m). The Hengduan Mountains run north–south, and the “Three Parallel Rivers” landscape is shaped by tectonic–fluvial synergistic processes. The Karst landforms of the Yunnan–Guizhou Plateau and the broom-shaped mountains in western Yunnan jointly construct a stepped three-dimensional habitat, providing unique spatial carriers for species diversity. The vertical zonation of climate is complete, covering tropical to alpine cold zones, with MAT ranging from 6 °C to 24 °C and mean annual precipitation (MAP) between 600 mm and 2300 mm. Distinct dry and wet seasons are driven by monsoon climate and topography, which jointly promote climate fluctuations and facilitate species migration, isolation, differentiation, and rapid speciation [66]. The triple coupling effects of “geography–geology–climate” make Yunnan a natural laboratory for biodiversity evolution research.

### 4.2. Sample Collection and Leaf Functional Trait Measurements

**Figure 6:**
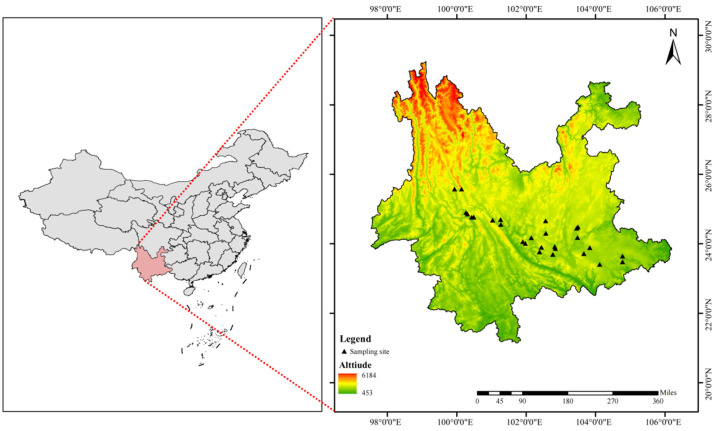
Distribution of sampling sites for *Polyspora chrysandra*. The base map was sourced from the China Standard Map Service System without modification. Map approval number: GS(2021)5443.

In this study, leaf specimens of mature and healthy plants of *P. chrysandra* were systematically collected from August to October 2024 in typical areas of Dali Bai Autonomous Prefecture, Honghe Hani and Yi Autonomous Prefecture, Chuxiong Yi Autonomous Prefecture, Yuxi City, Kunming City and Wenshan Zhuang and Miao Autonomous Prefecture in Yunnan Province (Figure 6, Table S5). The sampling campaign spanned 28 geographically dispersed sites, rigorously adhering to fundamental ecological sampling principles. Adjacent sampling points between populations were separated by a minimum distance of 10 km, with the exception of regions subject to significant geographical isolation. Within each population, individual samples were collected at least 5 m apart [62,67]. The sampling strategy accounted for the species’ natural distribution patterns and physical barriers (e.g., mountain ranges, rivers) to maximize spatial heterogeneity while ensuring ecological representativeness. The detailed sampling protocol was implemented as follows: At each of the 28 sampling sites, ten healthy, fully grown adult plants were carefully selected for sample collection. Thirty mature, functional leaves that had been fully exposed to sunlight throughout the day were randomly harvested from various positions on the outer periphery of the plant canopy. This process resulted in the collection of samples from 280 individual plants. For each plant, three biological replicates were established, yielding a total of 840 leaf samples and 25,200 individual leaves. All leaves were required to meet strict morphological criteria: fully expanded with intact laminas and without mechanical damage, pest infestation, foliar lesions, or herbivory marks. Post-collection, leaves from each site were pooled, immediately flash-frozen in liquid nitrogen, and transported to the laboratory under cryogenic conditions.

In this study, LFTs including SLA, LA, LDMC, LT, LWC, and CHL were selected as basic measurement traits following the Leaf Economic Spectrum (LES) theory, which identifies these traits as key indicators of plant resource acquisition–conservation strategies [63]. Considering the unique leaf morphological characteristics of *P chrysandra* (e.g., serrated margins, leathery texture) and the importance of petiole traits in determining leaf spatial positioning, mechanical support efficiency, and resource investment strategies, extended traits such as LSF, LSD, PL, and PD were included based on their demonstrated associations with environmental adaptation. To ensure reliable trait measurements, observer error was controlled through standardized protocols, personnel training, and pre-experiment validation. Protocols adhered to published guidelines [68,69], with explicit criteria defined for all 20 LFTs to ensure uniformity. Three trained researchers oversaw measurements to minimize variability. Prior to formal data collection, a pilot experiment was conducted using 20 standard leaves (representing diverse sizes/morphologies), with the three researchers conducting independent preliminary measurements; formal experiments commenced only after confirming inter-observer error < 5%. Leaf samples were hydrated with deionized water and equilibrated at 5 °C in a climate chamber for 12 h to standardize water status before measurement. All LFTs were quantified with 5 biological replicates for statistical power, and detailed methods are provided in Table S6.

### 4.3. Acquisition of Environmental Factor Data

GC data, including longitude (Lon), Lat, and Alt, were recorded using a high-precision GPS receiver at each sampling site. Because the Earth’s spherical geometry imposes circularity and periodicity on longitudinal data, treating Lon as a linear predictor could introduce spatial autocorrelation artifacts in statistical models. To address this, we applied trigonometric transformations (Lon→sin_Lon, cos_Lon) to preserve circular continuity while eliminating edge-effect biases in geospatial analyses [70]. Additionally, to confirm the effective control of spatial autocorrelation in the model, Global spatial autocorrelation of all 20 LFTs was evaluated using Geary’s C. A k-nearest-neighbor spatial weights matrix (k = 5, row-standardized) was constructed from the sampling coordinates. Geary’s C was calculated for each trait vector, with significance assessed via 999 random permutations. To control for multiple comparisons, *p*-values were adjusted using the Benjamini–Hochberg FDR procedure. All analyses were conducted in R 4.4.2 with the “sf” and “spdep” packages. Following Benjamini–Hochberg FDR correction, none of traits exhibited significant spatial autocorrelation (Geary’s C, *p*._adj_ ≥ 0.05, Table S7). Consequently, non-spatial models were employed for all traits without further spatial adjustments.

For CFs, data was taken from Climate’AP software (v3.10, https://web.climateap.net/), using average data from 2013 to 2022. This software leverages global meteorological station observations as baseline data and employs dynamic local downscaling techniques to transform it into a scale-free format. By employing bilinear interpolation and dynamic local regression, the climate data is downscaled to scale-free point data. This process generates high-precision climate datasets for any location in the Asia-Pacific region across various time scales (monthly, seasonal, annual, and future) based on latitude, longitude coordinates, and altitude [71], and the applicability of this dataset has been verified by relevant studies [72,73]. Compared with WorldClim2.1 (https://www.worldclim.org/), the most commonly used global climate dataset (with a maximum resolution of 1 km), ClimateAP further downscaled the data, combined with topographic correction and high-density site assimilation, to achieve a resolution of ≤250 m and a daily–monthly dual-scale output. In independent verification, the mean absolute error (MAE) of the monthly mean temperature was reduced by about 8%, and the precipitation MAE was reduced by about 35%. Compared with the original baseline data, the accuracy of the output climate data was significantly increased [74]. This study extracted 10 climatic variables, namely MAT, MWMT, MCMT, AHM, DD_5, DD_0, MAP, HRE, CMD, and RH, from latitude, longitude, and elevation data of species sampling sites. AI and PET were acquired from global raster data accessible via CGIAR-CSI (http://cgiarcsi.community). Utilizing the latitude and longitude coordinates of the sampling sites, ArcGIS 10.4 was employed to extract these variables from the Global-AI_PET_v3 database (Version 3) of the Global Aridity Index and Potential Evapotranspiration Database, curated by the Chinese Academy of Sciences Plant Data Center (https://www.plantplus.cn/).

SP data were acquired from the Harmonized World Soils Database (HWSD, v2.0; https://www.fao.org/soils-portal/en/, accessed on 28 February 2025) through the WheatA Agro-Meteorological Platform (v1.67d, https://wheata.cn/), with spatial resolution of 30 arc-seconds (~1 km). The dataset provides depth-weighted averages for two horizons: topsoil (0–30 cm) and subsoil (30~100 cm), including 11 physicochemical parameters: Gravel, Sand, Silt, Clay, RBD, OCC, pH, CEC, CECC, BS, and TEB.

UVRFs were obtained from the Global UV-B Database (https://www.ufz.de/gluv/), with spectral range of 280~315 nm at 0.5° × 0.5° grid resolution. Six biologically relevant UV-B indices were calculated: UVB1, UVB2, UVB3, UVB4, UVB5, and UVB6.

### 4.4. Data Analysis and Processing

#### 4.4.1. Data Preprocessing and Variable Screening

(1) Data processing and research methodology. Initial data organization utilized WPS Office (version 12.1, Kingsoft Office Software Co., Ltd., Zhuhai, China). Subsequent processing employed the “dplyr” package in R 4.4.2, applying the “Pauta criterion” to exclude outliers and improve data analysis accuracy and reliability [75]. In this study, we followed similar research approaches [76] by using CV and PI to analyze and describe the variation characteristics of LFTs. The calculation methods for these two indices were based on the formulas provided in the referenced literature [76]. CV is a statistical measure that quantifies the degree of dispersion in the data, reflecting the extent of variation in traits within a population or sample. The PI, on the other hand, is used to quantify the phenotypic plasticity of traits under different environmental conditions, reflecting the plant’s ability to adapt to environmental changes. Descriptive statistics for LFTs were analyzed with JASP 0.19.3 (JASP Team, University of Amsterdam, Netherlands). Data visualization was performed using GraphPad Prism 10.1.2 (Dotmatics, Boston, USA) and the “ggplot2” package in R 4.4.2.

A combined approach of PLS-SEM and RDA was employed to investigate the driving mechanisms underlying the variation in LFTs induced by environmental factors. The former aims to construct a causal path model, revealing the intrinsic relationships among various factors and capturing multi-level associations of direct and indirect effects; the latter focuses on quantifying the contribution and explanatory power of environmental variables to the variation in LFTs, with further clarification of the relative importance of each factor via HP.

**Figure 7:**
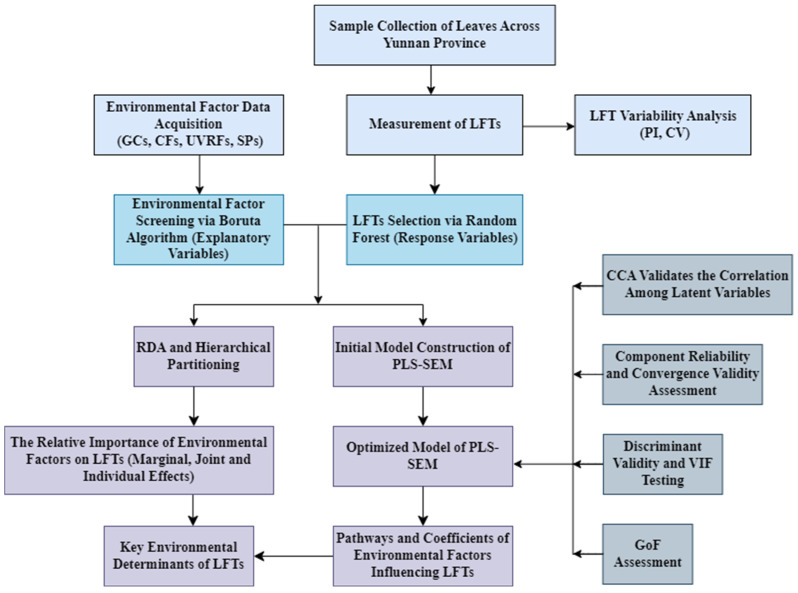
Research process flowchart.

The study’s research approach and workflow are depicted in Figure 7.

(2) Response variable screening. LFTs encompass numerous indicators, and including all in SEM risks multicollinearity, reducing model efficiency. To address this, we employed RF to select key LFTs based on their environmental explanatory power and variable importance. Feature importance was quantified via IncMSE, with higher values indicating stronger predictive contributions. To ensure the robustness of variable importance assessment, this study ran a RF model using 10-fold repeated cross-validation and applied bootstrap sampling (n = 1000) to test the significance of each variable’s IncMSE (*p* < 0.05). The “significance count” is defined as the number of times a variable’s IncMSE reached statistical significance in the 10 cross-validation runs. After removing variables with a significance count of 0, the remaining variables were ranked in descending order of significance count, and the top-ranked variables were included in the initial PLS-SEM model. This approach reduces the randomness of single analyses and prioritizes variables that are consistently important across multiple validations. To improve model stability and reliability, the number of decision trees for RF model was set to ntree = 1000, the number of repetitions for model training to nrep = 500, and num.cores = 4, minimizing random errors while considering computation time and resource limitations. This process primarily serves to reduce dimensionality and obtain a feature set with high explanatory power. Analyses were performed by the “rfPermute” package (R 4.4.2).

(3) Explanatory variable selection. Since RF can only provide variable importance rankings, it cannot fully assess the complex relationships and interactions between all variables and the response variable. Therefore, we used the indices selected by RF as the response variable and employed the Boruta algorithm to screen environmental factors (explanatory variables) to identify key factors (predictor variables) affecting leaf functional traits. The algorithm first generates shadow features by randomly permuting original features across observations, thereby creating an augmented feature set for importance testing. The significance of each original feature is evaluated using a RF classifier. During each iteration, if the importance score of an original feature exceeds the maximum score among the shadow features, it is considered to contribute significantly to the target variable and is retained; otherwise, it is eliminated. This iterative process continues until all features are clearly classified or the predefined maximum number of iterations is reached. The final selected environmental factors will serve as the foundational variables for constructing the structural equation model [77,78]. The confidence level is set at 0.01 (the default value), and the maximum number of iterations is set to 100 to ensure the reliability of the feature selection results. The Boruta algorithm offers distinct advantages over traditional methods. First, its global importance evaluation mechanism effectively addresses the local optimality issue associated with stepwise regression methods. Second, through the variable sampling strategy of RF, it can reduce the impact of collinearity among variables. Third, the inclusion of shadow variables provides a more robust threshold determination standard, eliminating the need for multiple comparison corrections (e.g., Bonferroni correction) required by univariate methods such as ANOVA and chi-square tests [79]. Furthermore, in comparison to Principal Component Analysis (PCA), the Boruta algorithm directly assesses the original variables, thereby avoiding the potential loss of practical meaning that may occur in the principal components derived from PCA. This enhances the interpretability of causal relationships among variables [80]. The Boruta algorithm was implemented using “Boruta” package in R version 4.4.2.

#### 4.4.2. Effects of Environmental Factors on Leaf Functional Traits and Their Relative Importance

In this study, RDA and HP were used to assess individual predictor contributions. RDA examines the overall variance between predictors and responses, offering a broad view of their interactions, while HP provides a more detailed decomposition, highlighting the specific impact of each predictor. In RDA, variable prescreening plays a crucial role in addressing the modeling challenges posed by high-dimensional environmental variables. Particularly when sample sizes are limited, the direct inclusion of numerous variables can lead to loss of degrees of freedom, biased parameter estimation, and collinearity issues. Although the Boruta algorithm is effective in removing redundant factors and capturing nonlinear ecological relationships during prescreening, its vulnerability to multicollinearity may leave residual correlations among the screened variables. Therefore, using a Variance Inflation Factor (VIF) criterion (VIF ≥ 10), we removed highly collinear variables, and the remaining environmental factors were incorporated into the RDA analysis. This approach enhances model robustness and ecological mechanism interpretation by simultaneously optimizing collinearity management and nonlinear feature extraction. Its effectiveness has been widely validated in biogeography and functional ecology research [81,82]. LFTs and environmental data were standardized and underwent DCA to determine the most appropriate ordination model. The overall explanatory power of the model was assessed via RDA with Constrained Ranking Analysis, whereas the explanatory contributions of individual variables remained unclear. To address this, HP theory was integrated into RDA to quantify the relative contribution of each explanatory variable to LFTs and compare the significance of environmental factors. The “vegan” package (R 4.4.2, R Core Team, Vienna, Austria, 2025) was used to perform VIF tests and RDA, with visualizations generated using Canoco5 (Biometris, Wageningen University & Research). HP were performed by the “rdacca.hp” [83] and “upset.hp” [84] packages.

#### 4.4.3. Construction and Evaluation of the PLS-SEM Model

(1) Initial Model Construction and Pathway Identification

SEM is a statistical framework used to construct, estimate, and evaluate causal relationships, accommodating both observable variables and unmeasurable latent variables. SEM comprises two primary approaches: covariance-based structural equation modeling (CB-SEM) and PLS-SEM. While CB-SEM focuses on parameter estimation, PLS-SEM excels in modeling complex causal relationships and offers distinct advantages in predictive accuracy. Unlike traditional linear structural relationship models, PLS-SEM enhances practical utility by simultaneously addressing reflective and formative model structures [85]. PLS-SEM is renowned for its efficacy in handling noisy data and missing values, making it well-suited for field observations where environmental factors are subject to substantial measurement errors [86]. It maintains robust predictive and explanatory capabilities while resolving measurement issues in small samples [30]. In exploratory studies with complex, under-researched variable relationships, PLS-SEM excels by uncovering structural patterns even with limited data. Compared to CB-SEM, it demonstrates superior robustness with non-normal distributions and small samples—a key factor in its adoption in ecology and environmental sciences [87,88]. In summary, although PLS-SEM is generally more suitable for small-sample studies, it offers greater flexibility in addressing data complexity and conducting exploratory research, thereby rendering it better suited for exploring complex relationships among latent variables. Based on the above considerations, following variable selection, this study selected PLS-SEM as the primary analytical method., using SmartPLS 4.1.0.3 (SmartPLS GmbH, Bönningstedt, Germany)—which supports a 20-case minimum [89]—to analyze environmental drivers of LFTs.

**Figure 8:**
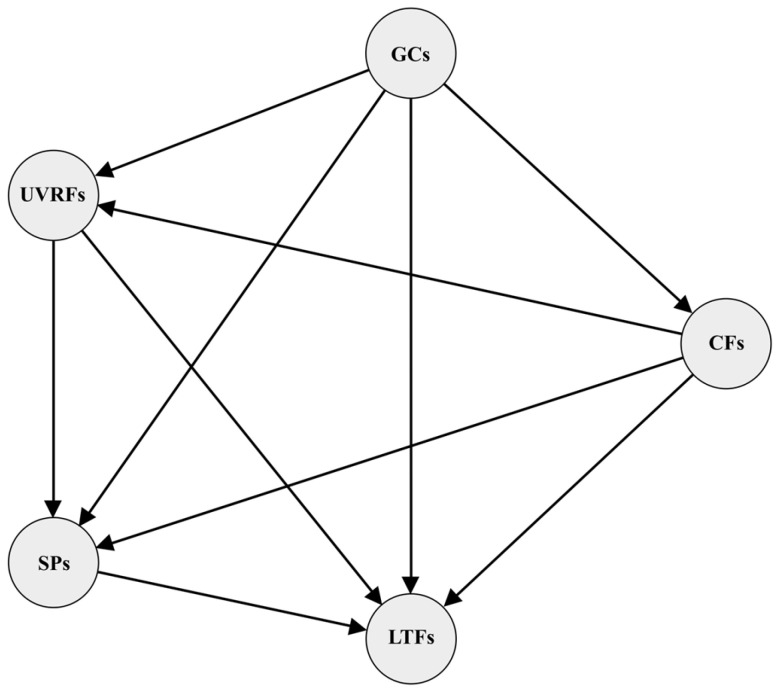
Potential path relationship among latent variables. Note: Circles represent latent variables, and single-headed arrows indicate the path relationships between latent variables.

The initial model posits six causal hypotheses: (a) GCs, CFs, SPs, and UVRFs directly affect LFTs (GCs→LFTs, CFs→LFTs, SPs→LFTs, UVRFs→LFTs); (b) GCs influence species distribution and ecological processes by altering CFs (e.g., moisture, temperature, light) and UVRFs, thereby indirectly affecting LFTs (GCs→CFs→LFTs, GCs→UVRFs→LFTs); (c) UVRFs alter SPs through photolysis, oxidation, and effects on microorganisms and vegetation, indirectly impacting LFTs (UVRFs→ SPs→LFTs); (d) GCs shape SPs, which in turn influence LFTs (GCs→SPs→LFTs); (e) GCs impact soil formation and nutrient distribution by modulating CFs, vegetation, and hydrothermal conditions, ultimately affecting LFTs (GCs→CFs→SPs→LFTs); (f) CFs indirectly regulate UVRFs by altering ozone thickness, atmospheric composition (e.g., aerosols, cloud cover), and surface albedo, thereby influencing LFTs (CFs→UVRFs→LFTs) (Figure 8).

To enhance model robustness and minimize uncertainty in PLS-SEM computations, this study employed CCA to systematically assess canonical correlations among observed variable groups. By constructing linear combinations between latent variable pairs, this approach elucidates the associative mechanisms across multiple variable groups. Its core advantage lies in treating variable groups as multidimensional entities for joint analysis, with canonical correlation coefficients (Rc) quantifying the explanatory power and effect magnitude of various factors on dependent variables. The study adopted a synergistic optimization strategy, integrating the B egorized into five latent groups: CFs, SPs, GCs, UVRFs, and LFTs (Figure 8). The interactions between latent variable groups were systematically analyzed using CCA, resulting in the construction of 10 bivariate association models. Rc was used to quantify the strength of associations between the groups, while path significance was evaluated through a *p*-value test based on 1000 permutations (α = 0.05). Rc and *p*-value are independent indicators: the former reflects the effect size (ranging from 0 to 1), while the latter indicates statistical reliability. This study employed a dual screening criterion to retain paths: (1) Rc ≥ 0.6 (strong association); (2) *p* < 0.05 (statistical significance). These pathways were incorporated into the SEM framework for parameter estimation and path validation. The CCA analysis was performed by IBM SPSS Statistics 27 (IBM Corp., Armonk, NY, USA).

(2) Model Evaluation

Building on the findings from the Boruta algorithm, RF feature selection, and CCA, this study employed PLS-SEM to perform factor analysis and path analysis. CR and AVE were used to assess the model’s construct reliability and convergent validity. Values of CR > 0.7 and Cronbach’s α > 0.7 indicated strong internal consistency, whereas AVE > 0.5 signaled acceptable convergent validity [90]. For discriminant validity assessment, beyond the Fornell–Larcker Criterion (i.e., the square root of a latent variable’s AVE must exceed its correlation coefficients with other latent variables) [90,91], this study additionally applied the Heterotrait–Monotrait Ratio (HTMT) method for cross-validation, given that PLS-SEM is prone to overestimating factor loadings and underestimating variable correlations, which may result in inflated AVE values [92]. According to the established literature, discriminant validity is confirmed when the HTMT value between latent variables is below 0.85 [93].

To address multicollinearity pertaining to observed variables, the VIF was used to spot collinearity issues among formative indicators. Specifically, each formative indicator variable should have a VIF value below 3.3 [94]. Reflective indicators are characterized by being influenced by latent variables, where the latent variable is the causal factor, and the indicators represent its observable manifestations. These indicators are expected to display consistent variation trends and strong correlations. Given the anticipated high inter-indicator correlation in reflective indicators, it is considered that VIF assessment is unnecessary [95,96]. In PLS-SEM analysis, the Q^2^ value is also a key indicator for evaluating the model’s predictive ability, and it is obtained by running the “Blindfolding” function, and the Q^2^value greater than 0 indicates acceptable predictive ability [97]. In PLS-SEM, significance testing for structural paths is conducted via bootstrapping, with a *p* -value less than 0.05 denoting statistical significance of the path coefficient [98]. For this research, the PLS algorithm was configured with a maximum of 5000 iterations and a stopping criterion based on a threshold for outer weight differences set at 10^−7^. To ensure reliability, 10,000 bootstrapping subsamples were utilized at a significance level of 0.05. The model fit was assessed using the Goodness of fit (GoF) measure: A GoF value of 0.36 or higher indicates excellent fit, a GoF value between 0.25 and 0.36 indicates moderate fit, and a GoF value less than 0.25 indicates poor fit [99]. All statistical analyses in PLS-SEM were conducted using SmartPLS (version 4.1.0.3, SmartPLS GmbH, Bönningstedt, Germany).

## 5. Conclusions

Through multivariate statistical analyses, this study revealed the ITV patterns of LFTs and their environmental drivers in natural populations of *P. chrysandra*—an ecologically important tree species in Yunnan Province. LFTs exhibited moderate ITV (CV range: 7.071%~25.650%), with SPs as the key determinants (31.51% contribution rate), exceeding the influence of CFs (11.04%). Among SPs, Silt showed the highest independent contribution and statistical significance (*p* < 0.05), and GCs (11.52%) and UVRFs (10.29%) further mediated trait divergence, with our study quantifying for the first time the direct effect of UVRFs on LFTs in this species. The interaction between RBD and CECC emerged as the most prominent coupled driver of trait variation. These findings address a critical knowledge gap in understanding how environmental factors shape intraspecific adaptive strategies of *P. chrysandra*, providing empirical evidence that SPs, rather than CFs, are the primary driver of ITV. Our results offer valuable data support for studies on environmental adaptation within the important genus *Polyspora*, particularly regarding soil-driven trait divergence and UV radiation effects. This work enhances our insight into ITV in ecologically key species and aids predictions of plant responses to environmental change.

## Figures and Tables

**Table 1:** LFTs’ characteristics of *P. chrysandra*.

LFTs	Max	Min	Mean ± SD	PI	CV (%)	Q1	Q3	Skewness	Kurtosis
PL	9.724	5.489	7.352 ± 1.243	0.436	16.906	6.472	7.981	0.668	−0.557
PD	2.835	1.824	2.248 ± 0.257	0.357	11.452	2.020	2.457	0.342	−0.678
LFW	1.975	0.816	1.261 ± 0.284	0.587	22.528	1.081	1.370	0.952	0.651
LDW	0.922	0.346	0.532 ± 0.125	0.625	23.544	0.453	0.589	1.335	2.365
CHL	72.771	44.281	59.895 ± 6.846	0.392	11.432	57.453	62.778	−0.279	0.137
LT	1.085	0.493	0.758 ± 0.136	0.546	17.995	0.650	0.859	0.149	−0.165
LSN	56.100	25.400	38.343 ± 7.567	0.547	19.734	32.725	43.425	0.584	−0.084
LSD	1.731	0.986	1.334 ± 0.204	0.430	15.272	1.223	1.477	0.100	−0.648
LL	18.453	9.075	12.530 ± 1.998	0.508	15.944	11.454	13.746	0.927	1.582
LW	5.435	3.305	4.300 ± 0.467	0.392	10.866	4.013	4.528	0.534	0.465
LA	65.600	19.052	36.928 ± 9.472	0.710	25.650	29.499	42.136	0.986	1.945
LP	42.149	21.723	29.129 ± 4.453	0.485	15.294	26.438	31.853	0.885	1.390
LWR	3.474	2.534	2.936 ± 0.280	0.271	9.522	2.691	3.178	0.215	−1.127
LSF	0.615	0.462	0.538 ± 0.042	0.249	7.861	0.512	0.576	0.239	−0.921
LWC	67.371	49.673	57.049 ± 4.034	0.263	7.071	54.506	59.119	0.581	0.688
LDMC	50.327	32.629	43.069 ± 3.986	0.352	9.255	41.047	45.494	−0.677	0.955
SLA	117.573	45.608	72.168 ± 16.086	0.612	22.292	59.925	78.523	1.129	1.514
LMA	0.022	0.009	0.015 ± 0.003	0.591	19.978	0.013	0.017	0.108	0.121
LSI	0.029	0.015	0.021 ± 0.003	0.483	16.098	0.019	0.023	0.343	0.115
LTD	0.303	0.119	0.207 ± 0.045	0.607	21.941	0.179	0.235	0.199	−0.130

Notes: Abbreviations used in the table are defined as follows, SD: Standard Deviation; PI: Phenotypic plasticity index; CV: Coefficient of Variation; Q1: First quartile (25th percentile); Q3: Third quartile (75th percentile).

**Table 2:** CCA and path coefficients among latent variables.

Latent Variables Group	Canonical Correlation Coefficients (Rc)	Eigenvalue	*p*-Value	Wilk’s	DF
GCs-CFs	0.932	6.608	0.000 ***	0.046	54.225
GCs-SPs	0.627	0.646	0.259	0.446	55.613
CFs-SPs	0.676	0.844	0.529	0.250	70.000
GCs-LFTs	0.853	2.669	0.001 **	0.066	43.371
CFs-LFTs	0.905	4.528	0.054	0.020	70.881
SPs-LFTs	0.878	3.380	0.004 **	0.019	65.728
GCs-UVRFs	0.963	12.820	0.000 ***	0.011	64.005
CFs-UVRFs	0.990	49.445	0.000 ***	0.000	60.210
UVRFs-SPs	0.977	20.686	0.000 ***	0.000	64.315
UVRFs-LFTs	0.999	647.935	0.193	0.000	19.022

Significance levels: ** *p* < 0.01, *** *p* < 0.001.

**Table 3:** PLS-SEM model quality assessment results.

	Cronbach’s α	CR	AVE	R^2^	Q^2^
CFs	0.954	0.989	0.881	0.510	0.198
GCs	0.845	0.933	0.754	—	0.452
LFTs	0.731	0.930	0.670	0.226	0.223
SPs	0.796	0.790	0.554	0.181	0.147
UVRFs	0.933	0.934	0.833	0.335	0.409

## Data Availability

All necessary data used to evaluate the conclusions in the manuscript are either included in the article itself or Supplementary Materials. Further inquiries can be directed to the corresponding author.

## Supplementary Materials

The following supporting information can be downloaded at: https://www.mdpi.com/article/10.3390/plants14192953/s1, Table S1, The importance values and significance levels of IncMSE for environmental factors; Table S2, Results of independent variable selection using Boruta algorithm; Table S3, Results of cross-loading analysis; Table S4, Results of discriminant validity analysis; Table S5, Latitude and longitude information of the sampling point of *P. chrysandra* across Yunnan Province; Table S6, Detailed methods for measuring leaf functional traits; Table S7, Geary’s C test results for spatial autocorrelation analysis; Figure S1, PI and CV for LFTs in *P. chrysandra*.
