# Supplementary material for: Intraspecific Variation and Environmental Determinants of Leaf Functional Traits in Polyspora chrysandra Across Yunnan, China
Source: Plants (Basel). 2025 Sep 23;14(19):2953. doi: 10.3390/plants14192953 (PMC12525973; doi:10.3390/plants14192953)
Supplement: Supplementary file 1 [file plants-14-02953-s001.zip › Table S1.pdf]

Table S1 The importance values and significance levels of IncMSE for environmental factors

Environmental factors	Increase in Mean Squared Error（IncMSE, %）																			
	PL	PD	LFW	LDW	CHL	LT	LSN	LSD	LL	LW	LA	LP	LWR	LSF	LWC	LDMC	SLA	LMA	LSI	LTD
Clay	-1.321	0.911	5.372	0.635	-0.134	-0.401	2.448	-1.730	4.811**	9.893*	7.668**	5.172*	0.465	-3.552	3.594**	4.779*	1.166	1.767	1.580	4.887*
OCC	-1.334	2.218	5.206	2.920	4.596	6.688	-2.309	-2.252	5.642*	7.548*	9.139*	7.849**	-3.483	-5.308	6.334*	6.163*	4.776**	2.953	-0.260	1.562
RBD	-1.041	1.042	5.566*	3.065	2.121	-2.684	0.023	-0.557	2.874	7.434**	6.048**	4.160*	-1.873	-1.903	7.398**	7.435*	5.200*	3.108	-0.462	0.958
TEB	-1.887	0.235	2.456	1.154	1.347	-2.128	-1.483	-4.251	4.949	5.089*	6.541*	5.602*	-0.985	-0.130	6.452*	8.000*	5.478*	4.863**	-1.265	2.647
Silt	-3.152	-0.025	1.377	-1.155	0.398	2.734	-1.735	-3.179	2.025	5.507*	5.681*	4.386	-3.860	-2.411	7.831**	7.602**	4.824*	4.994**	2.253	1.345
CECC	-0.886	0.781	6.929**	-0.089	2.726	-1.793	-2.421	-4.270	1.681	10.208**	4.519*	3.049	-3.662	-1.823	7.091**	6.421**	2.677	0.440	-0.711	-4.088
Sand	-1.452	0.120	6.222*	3.792	1.510	1.253	-1.603	-4.678	1.670	8.222*	6.820*	4.255	-1.117	-2.558	7.246*	8.806**	3.089	3.379	-0.410	-1.685
Alt	4.008*	-0.379	2.393	-0.962	-2.225	4.753	1.958	-3.929	4.301	1.789	1.540	3.694	6.801*	0.347	-0.458	0.199	3.887	12.905**	14.290**	4.336*
sin_Lon	2.217	0.065	0.866	2.265	2.146	11.486*	-2.401	-4.937	0.989	4.575	6.330*	2.476	-2.562	-1.274	6.303*	6.318*	4.373	2.485	3.422	2.533
Lat	-1.130	3.753	5.373*	5.052	-0.138	4.300	-1.311	-5.024	-0.254	7.187*	2.007	1.483	5.328*	3.729	0.089	0.168	-0.677	2.853	6.808	-0.087
DD_0	-1.781	-1.805	-2.006	-0.991	1.290	0.336	-0.780	-0.559	-1.267	-1.166	0.303	-1.675	1.516	-0.709	2.034	2.499*	-0.461	2.557*	3.252**	2.421
MAT	2.118	1.911	1.319	0.022	1.261	-2.301	1.992	-2.097	-0.044	-0.334	-0.152	2.248	3.958*	4.654*	1.639	0.957	3.324	5.287	6.442**	4.470
MCMT	3.201	0.480	-0.353	0.961	-0.711	0.384	3.136	-1.302	2.993	-1.372	0.873	1.667	2.930	3.165	4.591	3.455	4.605	7.123*	7.896*	6.589*
pH	-0.048	-0.469	1.848	-1.744	0.012	0.828	-2.162	-3.483	3.899	2.088	3.118	4.772*	-1.796	-2.093	2.850	4.563*	4.782**	3.480	1.636	3.471
UVB1	0.852	3.924	0.895	1.242	8.219*	2.616	-0.997	-1.935	4.944	3.542	3.139	4.907*	-2.003	-1.463	-0.831	0.870	-2.202	2.173	-0.171	5.049*
cos_Lon	-0.294	10.885**	-1.342	-2.658	1.433	10.139*	0.432	0.521	2.169	1.738	3.097	1.382	-1.447	-0.648	-0.595	2.127	0.580	1.031	-1.295	2.742
AHM	5.684*	-0.969	0.646	-1.792	3.851	17.661**	0.235	-2.693	0.128	-1.088	-0.262	0.002	-0.334	2.445	1.129	-1.375	-1.192	1.642	-1.252	3.271
DD_5	0.990	-0.179	1.084	-1.198	2.272	-2.063	4.449	-1.141	0.596	-1.414	1.414	1.498	1.032	3.987	0.113	1.585	1.432	4.377	8.206**	4.192

**Environmental factors**	**Increase in Mean Squared Error（IncMSE, %）**																			
	PL	PD	LFW	LDW	CHL	LT	LSN	LSD	LL	LW	LA	LP	LWR	LSF	LWC	LDMC	SLA	LMA	LSI	LTD
MAP	4.176	0.276	-2.650	-1.708	2.515	***6.955****	0.032	-4.167	-2.251	-0.838	-1.898	-2.210	1.090	0.032	-1.016	-0.194	-0.717	-0.576	-1.523	2.790
Gravel	-1.475	-0.404	-0.008	-2.825	***5.531****	-4.534	-3.319	-1.649	-1.505	1.138	0.921	1.038	-2.429	-2.371	-0.724	-2.397	-0.654	-0.436	-0.089	-0.983
UVB2	-0.489	-1.054	-1.069	-1.330	***7.137****	-0.172	-3.024	-2.586	-1.895	1.644	-0.689	-0.298	-1.912	-2.109	-0.608	0.064	-1.929	-3.251	-4.477	0.470
UVB3	0.897	2.206	-1.362	-1.085	***7.669****	0.866	-1.499	-3.685	-0.829	1.279	1.909	1.824	-1.519	-2.640	-1.107	-0.238	-2.151	-0.472	-2.557	1.900
UVB5	2.074	-0.155	0.483	-2.004	***8.991*****	3.725	-1.905	-3.611	0.709	2.084	-0.747	0.279	-4.060	-0.028	1.081	1.561	2.245	0.637	-2.920	5.060
UVB6	3.581	3.003	-0.819	-1.747	***4.942****	0.972	-2.720	-1.698	3.507	2.196	4.404	3.615	0.052	-0.273	-1.798	-2.061	-2.664	-4.108	-1.076	1.442
AI	-1.486	3.928	4.174	2.380	-0.447	2.551	0.094	1.975	0.665	2.617	1.008	-1.913	-1.881	-1.276	-0.590	-1.821	-0.690	-0.423	-3.191	-2.883
CMD	2.138	2.410	1.349	0.178	0.829	4.463	-1.365	0.360	-2.287	-0.791	-1.398	-3.212	1.033	1.052	2.807	0.743	-2.937	-0.635	-3.199	-0.937
HRE	2.535	1.217	-1.438	1.187	-0.698	2.290	-0.452	-3.287	-1.388	-1.552	0.842	-0.175	-2.323	-1.347	-0.783	-1.207	-1.731	-2.507	1.152	3.895
MWMT	1.550	-0.948	0.447	-0.915	1.443	1.465	0.376	-1.640	1.631	0.487	0.508	2.103	3.498	2.576	0.497	0.643	2.268	3.236	5.493	2.852
RH	-2.463	1.999	-0.146	-0.756	0.595	-1.549	-0.311	-0.361	1.075	0.883	-0.142	-2.402	-0.092	1.931	-2.326	1.932	-1.220	0.924	1.751	1.544
PET	-4.160	4.771	-0.220	0.310	3.693	2.206	-1.274	-2.790	0.908	0.918	3.886	-1.224	-3.774	-4.395	-1.033	2.297	0.748	0.831	1.694	2.205
BS	-1.281	-0.754	0.652	-0.843	-0.010	0.926	-2.675	-3.434	-0.550	1.741	1.734	0.081	-1.998	-3.149	1.073	-1.343	-2.792	-0.911	0.388	0.434
CEC	-1.361	-1.302	0.851	2.747	0.182	1.085	-0.815	-2.109	2.920	4.226	3.108	2.921	-0.270	-2.087	-1.899	-3.499	-1.465	0.377	-1.314	-0.876
UVB4	-0.033	3.214	-2.551	-2.952	3.082	-0.879	0.970	-4.086	2.836	1.211	4.113	3.888	0.462	0.211	-0.111	-1.731	-0.408	-2.097	0.784	1.074

Note: Indicators presented in italicized and bolded format denote statistical significance. Significance levels: ** $p < 0.01$, * $p < 0.05$.
