# Supplementary material for: Intraspecific Variation and Environmental Determinants of Leaf Functional Traits in Polyspora chrysandra Across Yunnan, China
Source: Plants (Basel). 2025 Sep 23;14(19):2953. doi: 10.3390/plants14192953 (PMC12525973; doi:10.3390/plants14192953)
Supplement: Supplementary file 1 [file plants-14-02953-s001.zip › Table S2.pdf]

**Table S2** Results of independent variable selection using Boruta algorithm

| **Independent variables (Environmntal factors)** | **Dependent variables (LTFs)** | **Independent variable selected by Boruta （Confirmed）** | **Ranked by vote Count** |
|---|---|---|---|
| GCs | LDMC | Lat，sin_Lon | sin_Lon（**8**）<br>Lat（**4**）<br>Alt（**3**）<br>cos_Lon（**2**） |
| | LWC | sin_Lon | |
| | LW | cos_Lon, sin_Lon | |
| | LA | sin_Lon | |
| | LP | sin_Lon | |
| | CHL | / | |
| | SLA | Alt，sin_Lon | |
| | LMA | Alt，Lat，sin_Lon | |
| | LSI | Alt，Lat | |
| | LT | cos_Lon，sin_Lon | |
| | LFW | Lat | |
| | LTD | / | |
| UVRFs | LDMC | UVRF1 | UVB1（**4**）<br>UVB3（**2**）<br>UVB5（**2**）<br>UVB2（**1**）<br>UVB6（**1**）<br>UVB4（**0**） |
| | LWC | UVRF1，UVRF3，UVRF5 | |
| | LW | UVRF1 | |
| | LA | / | |
| | LP | / | |
| | CHL | UVB1，UVB2，UVB3，UVB5，UVB6 | |
| | SLA | / | |
| | LMA | / | |
| | LSI | / | |
| | LT | / | |
| | LFW | / | |
| | LTD | / | |
| CFs | LDMC | / | MCMT（**4**）<br>DD_5（**3**）<br>AHM（**3**）<br>MWMT（**3**）<br>MAP（**1**）<br>DD_0（**1**） |
| | LWC | / | |
| | LW | / | |
| | LA | / | |
| | LP | / | |
| | CHL | AHM | |
| | SLA | DD_5，MCMT，MWMT | |
| | LMA | MCMT，MWMT | |
| | LSI | DD_5，DD_0，MCMT，MWMT | |
| | LT | AHM，MAP | |
| | LFW | / | |
| | LTD | DD_5，AHM、MAP，MCMT | |

| Independent variables (Environmntal factors) | Dependent variables (LTFs) | Independent variable selected by Boruta （Confirmed） | Ranked by vote Count |
|---|---|---|---|
| SPs | LDMC | TEB， CECC， OCC， RBD， Silt， Sand | OCC（8）<br>TEB（7）<br>RBD（7）<br>Silt（7）<br>Sand（6）<br>CECC（6）<br>Clay（5）<br>pH（1） |
| | LWC | TEB， CECC， OCC， RBD， Silt， Sand | |
| | LW | TEB，CECC，OCC、RBD，Clay，Silt，Sand | |
| | LA | TEB，CECC，OCC，RBD，Clay，Silt，Sand | |
| | LP | TEB， pH， OCC， RBD， Clay， Silt | |
| | CHL | / | |
| | SLA | TEB， pH， RBD， Silt， Sand | |
| | LMA | TEB， CECC， pH， RBD， Clay， Silt， Sand | |
| | LSI | Clay | |
| | LT | OCC | |
| | LFW | CECC， OCC， RBD， Clay， Silt， Sand | |
| | LTD | OCC | |

**Note:** The symbol " / " indicates that all independent variables were classified as either "Rejected" or "Tentative" by the Boruta algorithm when this metric was used as the dependent variable.
