# Supplementary figures and images for: Intraspecific Variation and Environmental Determinants of Leaf Functional Traits in Polyspora chrysandra Across Yunnan, China
Source: Plants (Basel). 2025 Sep 23;14(19):2953. doi: 10.3390/plants14192953 (PMC12525973; doi:10.3390/plants14192953)

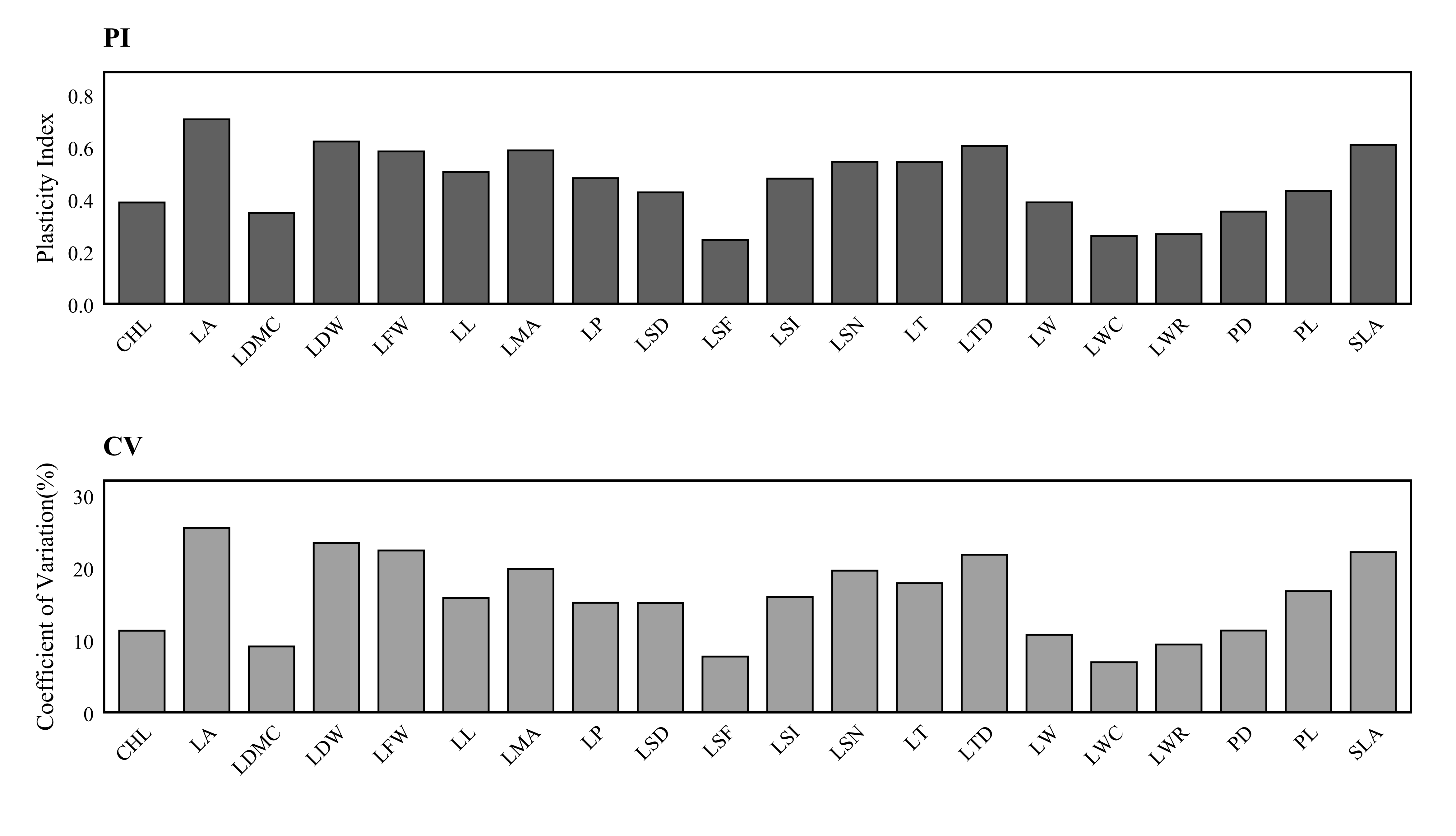

Supplement: Supplementary file 1 [file plants-14-02953-s001.zip › Figure S1.tif]
